# A rare cause of shock in cases of refractory hypotension, hypoproteinaemia and haemoconcentration

**DOI:** 10.1016/j.clinme.2025.100550

**Published:** 2026-01-05

**Authors:** Omer Elhassan, Scott Williams, Frank Joseph

**Affiliations:** a Department of Medicine, Countess of Chester Hospital, Liverpool Road, Chester, England, CH2 1UL, UK; b School of Medicine, University of Chester, Parkgate Rd, Chester, England, CH1 4BJ, UK

**Keywords:** Hypotension, Hypoproteinaemia, Haemoconcentration, Sepsis, Idiopathic systemic capillary leak syndrome, Clarkson’s disease

## Abstract

This case report describes a woman in her mid-40s of European ancestry presenting with recurrent profound hypotension, hypoproteinaemia and haemoconcentration. She first presented following administration of a Japanese encephalitis vaccine. The initial differential diagnoses included sepsis or an anaphylactic reaction to the vaccine. No evidence of allergy or infection was found during extensive investigation including a lumbar puncture. Following a second similar presentation, she was diagnosed with idiopathic systemic capillary leak syndrome (ISCLS). Her presentation was highly atypical due to the high frequency of episodes encountered (34 over a near 6-year period). A variety of triggers for episodes were identified including COVID-19 infections, influenza infection and vaccinations. We discuss her clinical presentation, investigations, ISCLS episode triggers and management. This highlights the benefits of intravenous immunoglobulins (IVIg), both for prophylaxis and for the acute treatment of ISCLS episodes.

## Introduction

Idiopathic systemic capillary leak syndrome (ISCLS) is a rare, life-threatening disorder most frequently reported in relatively young European individuals. ISCLS was first described by Clarkson *et al* in 1960 in a 34-year-old woman, whose episodes of severe hypotension correlated with menstruation.^1^ Here we describe a similar presentation in a woman in her mid-40s (for background, see Supplementary file).

### Case presentation

A European female in her mid-40s presented to the emergency department with a 1-day history of nausea and vomiting following administration of a Japanese encephalitis vaccine. Her Glasgow Coma Scale was 15/15, with an elevated heart rate, low blood pressure (BP) and hypovolaemia. Intravenous (IV) antibiotics (piperacillin/tazobactam) and fluid resuscitation was commenced, treating as septic shock, with a septic screen and urine output monitoring. After 7 L of crystalloid IV fluid were administered over 7 h, she remained hypotensive with oliguria and an acute kidney injury (AKI).

Laboratory findings (investigations - see Supplementary file) showed a raised white cell count with a neutrophilia and confirmed an AKI biochemically with a metabolic acidosis. Repeat bloods showed the most striking biochemical abnormality of a sudden drop in her albumin to 6 g/L from 27 g/L with haemoconcentration. Our patient developed anasarca with severe shock, requiring immediate transfer to the intensive care unit (ICU) for inotropic support. Triple inotropes with noradrenaline, vasopressin and dobutamine increased her BP from 70/40 mmHg. Hydrocortisone 100 mg IV was given as the patient was *in extremis*, and the patient was intubated and ventilated due to agitation and confusion. Antibiotic therapy was escalated to include IV meropenem, clindamycin, vancomycin and aciclovir for broad antimicrobial cover. A computed tomography (CT) scan of the thorax, abdomen and pelvis showed a right upper lobe segmental pulmonary embolus and ischaemic colitis in the ascending colon. An echocardiogram showed a small rim of pericardial effusion posteriorly. The patient’s BP and urine output improved. She had generalised oedema, responding to a short course of IV diuretics, and was extubated. She was discharged home after 10 days.

### Differential diagnosis

The differential diagnosis included infection with sepsis, an anaphylactic reaction to a vaccine, or an immunological disorder. Investigations, including an infection screen with blood cultures, urine cultures, urine legionella and pneumococcal antigen, cerebrospinal fluid analysis, human immunodeficiency virus (HIV), hepatitis B virus, hepatitis C virus, a vasculitis screen and serum electrophoresis, all yielded negative results with no evidence of infection.

### Initial management and prognosis

Following a second similar presentation, the patient was diagnosed with idiopathic systemic capillary leak syndrome (ISCLS). Prophylactic treatment was commenced with terbutaline 5 mg four times a day, with side effects of tachycardia with palpitations, and was discontinued due to lack of efficacy. Oral theophylline was commenced with dose titration to 175 mg twice a day to achieve a therapeutic level, and was discontinued due to lack of efficacy. Montelukast 10 mg once a day was also commenced, then discontinued with no benefit. The patient experienced 6 leaks over 10 months prior to commencing IV immunoglobulin (IVIg). Monthly IVIg is an effective prophylactic treatment for preventing acute episodes of ISCLS.^2^ After commencing IVIg therapy, the patient did not experience an episode over a 9-month remission period. IVIg was continued regularly on the medical outpatient unit, with a good response to standard monthly IVIg therapy at a dose of 2 g/kg monthly. IVIg proved of significant benefit in reducing the frequency and severity of ISCLS episodes, avoiding hospital admissions and improving the patient’s quality of life. National IVIg panel approval was sought for ongoing IVIg treatment. The prognosis of ISCLS is unclear, but it has been demonstrated in the literature that prophylactic treatment with IVIg or a beta 2 agonist can improve 5-year survival from 20% to 85%.^3^

### Case progression and outcome

In total, the patient suffered 34 episodes of ISCLS over a near 6-year period. Each acute episode of ISCLS in our patient was defined by hypotension with a systolic BP less than 90 mmHg, hypoalbuminaemia and haemoconcentration.^4^ Her disease course (Fig. 1, see Supplementary file) followed five distinct periods separated by the difference in frequency of episodes (from presentation and diagnosis, first remission, first relapse, second remission and second relapse). Placement of a peripherally inserted central cannula (PICC) line was essential to provide reliable IV access. Training in the sterile process was provided to the patient and her next of kin to allow for IVIg infusions at home. Once-monthly IVIg helped to prevent ISCLS episodes and reduce hospital admissions. Collaboration with another NHS hospital allowed the patient to receive monthly IVIg while on holiday in the UK. Treatment of an acute episode of ISCLS in our patient included crystalloid IV fluid resuscitation and urgent early referral to the ICU for inotropes. IV crystalloid 1 L was given immediately, with further 250–500 mL boluses determined by BP/fluid balance. Noradrenaline was the initial vasopressor used, titrated with ICU care. Any clinical deterioration during IV fluid resuscitation including an increasing NEWS2 score, hypotension or oliguria were used as triggers for ICU admission. We also placed an alert in the medical notes to alert any admitting team to inform the ICU, because managing our patient on ICU for the first 48 h of each episode was the most appropriate place of care. This allowed close monitoring of central venous and arterial pressure which guided fluid resuscitation, reducing the risk of cardiogenic pulmonary oedema. Cardiogenic pulmonary oedema did occur during the recovery phase and was treated with IV diuretics. The patient was stepped down to the acute medical unit when clinically improving prior to discharge. A high dose of IVIg of 70 g was also effective in treating acute ISCLS. It was time critical to have the IVIg ready for administration immediately in the ED resuscitation room. ISCLS episodes often coincided with menstruation. We commenced the oral contraceptive pill desogestrel, but this did not reduce episodes and was stopped. Monoclonal gammopathy of undetermined significance (MGUS) was diagnosed after 5 years. The next detected COVID-19 infection triggered a final ISCLS episode, leading to the patient’s death nearly 6 years after her diagnosis. The patient died from refractory shock that was unresponsive to IV fluid resuscitation, inotropes and IVIg during the final episode. Severe hypotension and pulmonary oedema led to multi-organ failure.

## Discussion

Unusually for them, the patient felt very fatigued in the year prior to presenting, suggesting that they could have been suffering from undiagnosed ISCLS, and the Japanese encephalitis vaccine administration triggered a severe ISCLS episode. ISCLS episodes may be triggered by viral or bacterial infection, vaccinations, coincide with menstruation or have an unknown trigger.^2^ Infections were the most common trigger for our patient, with at least three episodes during infections. One episode occurred after confirmed influenza, and at least two episodes occurred after proven COVID-19, with possibly a further episode due to undiagnosed COVID-19. Interestingly, a recent case report this year described a man in his late 30s who presented with ISCLS following COVID-19 infection.^5^ He was diagnosed with a myeloproliferative neoplasm, suggesting that viral infections including COVID-19 may act as a trigger for ISCLS in patients with another underlying contributing factor including malignancy or a genetic predisposition. The precise pathophysiology of ISCLS remains unclear.^6^ Menstruation may have been a trigger for ISCLS episodes in our patient. The second relapse with increased ISCLS episodes occurred when the IVIg dose was reduced, suggesting that the clinical response to IVIg treatment was dose dependent. The patient had responded clinically to the higher dose, with a reduction in episodes during the second remission. IVIg is the most effective prophylactic long-term therapy to reduce ISCLS episodes, and also to treat acute episodes of ISCLS requiring hospitalisation.^4^ Continuing IVIg treatment is supported by the literature including Moyon *et al*, who demonstrated that withdrawal of IVIg is associated with increased mortality and higher rate of recurrence in patients with MGUS-associated ISCLS.^7^, ^8^ ISCLS with MGUS is a common finding and is a more severe form with lower survival.^2^ Our patient died shortly after detecting MGUS, with a severe episode leading to the patient’s death during a COVID-19 infection. This is consistent with a 2024 case report of a man in his mid-70s with recurrent ISCLS episodes and MGUS presenting with syncope due to recurrent hypotension.^8^ There is not a universal consensus definition of ISCLS.^2^^,^^4^^,^^9^ We used clinical judgement of systolic BP below 90 mmHg, with prodromal symptoms or an increasing National Early Warning Score 2 (NEWS2) with worsening vital signs to rapidly recognise episodes.^10^ The NEWS2, updated by the Royal College of Physicians (RCP) in 2017, helped to identify acute episodes.^10^ We had significant difficulties in obtaining immunology input to help manage this condition due to its rarity.

## Summary: Key learning points

1. ISCLS is a rare, life-threatening disease. Early recognition and treatment are essential.
2. Triggers for ISCLS episodes include viral infections, bacterial infections and vaccine administration. Episodes may correlate with menstruation.
3. IVIg is the most effective prophylactic long-term therapy to reduce episodes, and to treat acute episodes of ISCLS.
4. Further research on ISCLS will develop the most effective management strategies.

## CRediT authorship contribution statement

**Omer Elhassan:** Writing – review & editing, Writing – original draft. **Scott Williams:** Writing – review & editing, Writing – original draft. **Frank Joseph:** Writing – review & editing, Writing – original draft.

## Declaration of competing interest

The authors declare that they have no known competing financial interests or personal relationships that could have appeared to influence the work reported in this paper.

## Appendix Supplementary materials

Image, application 1
