# Supplementary material for: A rare cause of shock in cases of refractory hypotension, hypoproteinaemia and haemoconcentration
Source: Clin Med (Lond). 2026 Jan 5;26(2):100550. doi: 10.1016/j.clinme.2025.100550 (PMC12857328; doi:10.1016/j.clinme.2025.100550)

**Background**

Fewer than 500 cases of Idiopathic Systemic Capillary Leak Syndrome (ISCLS) have been reported in the medical literature including a small number in children (fourteen children over a six-year period).^3^ There are also now some reports of ISCLS in individuals of non-European ancestry including in individuals of Asian, African American, Hispanic, and Middle Eastern ancestry.^2^ There is a near equal proportion of cases in males and females (52% of cases are female) with no sex predominance.

There is often a failure to recognise the early clinical features of ISCLS including lethargy, abdominal pain, nausea, and vomiting with a significant delay in diagnosis. These symptoms represent the prodromal phase, which is one of three distinct clinical phases of ISCLS. This is followed by the fluid extravasation phase presenting with hypotension, haemoconcentration and hypoalbuminemia. This triad is indicative of the diagnosis of ISCLS although specific diagnostic criteria are lacking.^2^

The possible underlying pathophysiology of ISCLS is endothelial wall dysfunction, allowing extravasation of plasma and proteins into the interstitium.^1^ The aetiology of systemic capillary leak syndrome includes malignancy, infections, drugs, vaccines, and idiopathic cases (ISCLS) are seen with unknown aetiology.^2^ Episodes of ISCLS can vary in their clinical severity and frequency, with some patients experiencing one lifetime episode and others experiencing multiple frequent episodes.^2^ Patients present with plasma extravasation of up to 70% of total plasma volume into the extravascular space causing tachycardia, excessive thirst, and oliguria with an increased risk of venous thromboembolism due to haemoconcentration.^3^  Subsequent end organ hypoperfusion due to hypovolemia can result in catastrophic sequalae including renal or liver failure and hypoxic brain injury.^3^ If the patient survives a life threatening episode of ISCLS, during the recovery phase fluid is recruited back into the intravascular space. Therefore, the patient’s blood pressure, haematocrit and urine output returns to normal between episodes.^2,3^ A review of 30 cases of ISCLS over a six-year period found a mean duration of episodes of 3.8 days, with episode length ranging from 1 to 27 days.^3^ Laboratory findings in SCLS include low albumin levels, haemoconcentration, polycythaemia, and Monoclonal Gammopathy of Undetermined Significance (MGUS).^4^ The initial treatment of the acute ISCLS episode should be commenced immediately at presentation of hypotension and haemoconcentration in the resuscitation room of the Emergency Department (ED) with a seamless transition to the Intensive Care Unit (ICU). Intravenous (IV) isotonic crystalloid fluid resuscitation is administered with albumin to maintain vital organ perfusion and prevent a severe metabolic acidosis.^2,3^ Central Venous Pressure (CVP) and / or arterial line monitoring is used for guiding inotropic support, and steroids may be administered depending on the clinical response to treatment (including the vital signs of Blood Pressure (BP), Heart Rate, and Urine Output). Ongoing judicious IV fluid resuscitation followed by IV diuretics are required to prevent fluid overload during the recovery phase.^2,3,4^ IVIg significantly improves survival of patients with ISCLS when administered prophylactically for preventing ISCLS episodes.^5^

We are one of the first case reports to the best of our knowledge to describe the significant benefit of administering IVIg for the treatment of acute ISCLS episodes, in addition to their known beneficial role in prophylaxis. Other treatments which may be trialled separately or in combination for prophylaxis of episodes with a limited evidence base include Beta 2 agonists, theophylline, methylxanthine, and plasmapheresis.^2,3,5^

**Investigations:** (abnormal results in bold)

| Lab Test | 0 hour | 16 Hours | 30 Hours | Normal Range |
| --- | --- | --- | --- | --- |
| HAEMOGLOBIN (Hb) | **213** | 147 | 132 | 115-160 g/L |
| HAEMATOCRIT (HCT) | **0.65** | 0.45 | 0.39 | 0.34-0.46 |
| ALBUMIN | **27** | **6** | 33 | 32-46 g/L |
| TOTAL PROTEIN | 50 | 26 | - | 60-80 g/L |
|  |  |  |  |  |
| Lab Test | 0 hour | Normal Range |  |  |
| RED BLOOD CELLS | 7.5 | 3.5-5.5 10*12/L |  |  |
| MEAN CORPUSCULAR VOLUME | 86 | 82.0 – 100.0 |  |  |
| MEAN CORPUSCULAR HAEMOGLOBIN | 28.4 | 26.0 – 32.6 g/dL |  |  |
| MEAN CORPUSCULAR HAEMOGLOBIN CONCENTRATION | 329 | 320-360 g/L |  |  |
| WHITE CELL COUNT | **24.7** | 4.0 – 11.0 10*9/L |  |  |
| PLATELETS | 332 | 120-400 10*9/L |  |  |
| NEUTROPHILS | **22.4** | 2.0-6.0 10*9/L |  |  |
| LYMPHOCYTES | 1.5 | 1.0 – 3.5 10*9 g/L |  |  |
| MONOCYTES | 0.6 | 0.18 - 0.86 10*9 /L |  |  |
| EOSINOPHILS | 0.0 | 0.0 – 0.46 10*9 g/L |  |  |
| SODIUM | 135 | 133-144 mmol/l |  |  |
| POTASSIUM | 4.8 | 3.5 – 5.0 mmol/l |  |  |
| STANDARD BICARBONATE (HCO_3_^-^) | 11 | 20-32 mmol/l |  |  |
| UREA | **9.5** | 2.0-6.5 mmol/l |  |  |
| CREATININE | **131** | 50-120 umol/l |  |  |
| CALCIUM | 1.76 | 2.10 – 2.70 mmol/l |  |  |
| BILIRUBIN | 10 | 3-20 umol/l |  |  |
| ALANINE TRANSAMINASE | 13 | 8-45 U/L |  |  |
| ALKALINE PHOSPHATASE | 58 | 46-148 U/L |  |  |
| GLOBULIN | 23 | 23/39 g/L |  |  |
| ADJUSTED CALCIUM | 2.61 | 2.1-2.7 mmol/l |  |  |
| CREATINE KINASE | 90 | 26 – 140 U/L |  |  |
| PROLACTIN | 514 | 82-524 mu/L |  |  |
| AMYLASE | < 10 | 12-122 U/L |  |  |
| HUMAN CHORIONIC GONADOTROPIN | <0.5 | - |  |  |
| C-REACTIVE PROTEIN | 8 | < 10 mg/L |  |  |
| LACTATE | **4.5** | 0.6 – 2.5 mmol/L |  |  |
| pH | **7.29** | 7.35 – 7.45 |  |  |


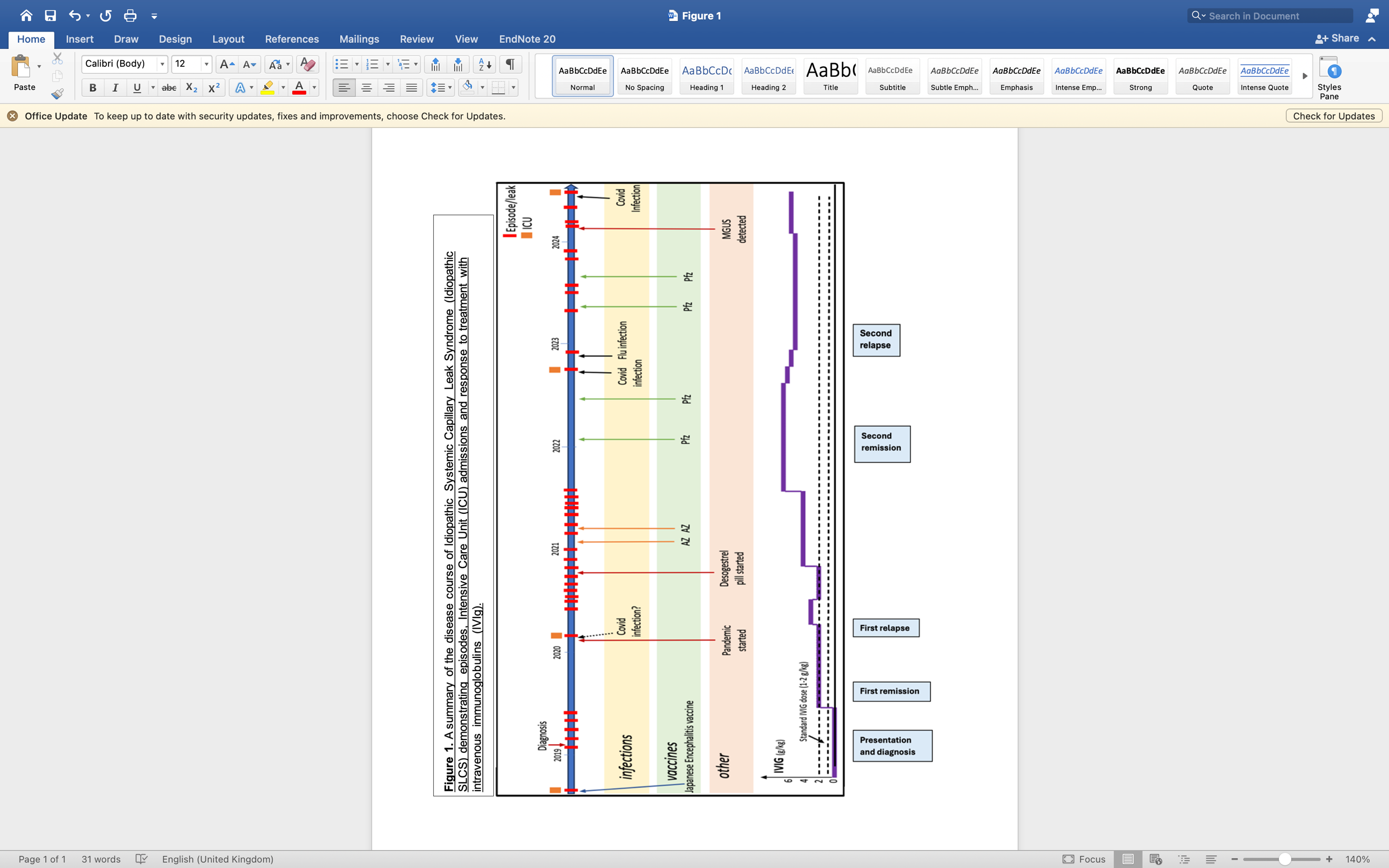

Supplement: Supplementary file 1 [file mmc1.docx]
